# Geographical information system and spatial–temporal statistics for monitoring infectious agents in hospital: a model using *Klebsiella pneumoniae* complex

**DOI:** 10.1186/s13756-021-00944-5

**Published:** 2021-06-16

**Authors:** Priscila Pinho da Silva, Fabiola A. da Silva, Caio Augusto Santos Rodrigues, Leonardo Passos Souza, Elisangela Martins de Lima, Maria Helena B. Pereira, Claudio Neder Candella, Marcio Zenaide de Oliveira Alves, Newton D. Lourenço, Wagner S. Tassinari, Christovam Barcellos, Marisa Zenaide Ribeiro Gomes, on behalf of Nucleus of Hospital Research Study Collaborators, Vitoria Pinson Ruggi Dutra, Maxuel Cassiano da Silva, João Pedro Silva Tonhá, Luciana Sênos de Mello, Murillo Marçal Castro, Yann Rodrigues Mathuiy, Amanda Aparecida da Silva Machado

**Affiliations:** 1 Laboratório de Genética Molecular de Microrganismos, Instituto Oswaldo Cruz - Fundação Oswaldo Cruz, Avenida Brasil, 4365, Pavilhão Leônidas Deane, 6º andar, sala 607, Rio de Janeiro, RJ 21040-900 Brazil; 2 Department of Engineering, Hospital Federal Dos Servidores Do Estado (HFSE), Rio de Janeiro, RJ Brazil; 3 Laboratory of Microbiology, HFSE, Rio de Janeiro, RJ Brazil; 4 Hospital Infection Control Committee, HFSE, Rio de Janeiro, RJ Brazil; 5 Admitting Office, HFSE, Rio de Janeiro, RJ Brazil; 6 Department of Mathematics, The Federal Rural University of Rio de Janeiro, Rio de Janeiro, RJ Brazil; 7 Institute of Scientific and Technological Communication and Information in Health, FIOCRUZ, Rio de Janeiro, RJ Brazil; 8 Present Address: Rio de Janeiro State University, Rio de Janeiro, Brazil; 9 Present Address: Evandro Chagas National Institute of Infectious Diseases, FIOCRUZ, Rio de Janeiro, Brazil

**Keywords:** Surveillance, Hospital infection, Antimicrobial resistance, *Klebsiella pneumoniae*, Time series, Geographic information system, Spatial–temporal statistics

## Abstract

**Background:**

The emergence and spread of antimicrobial resistance and infectious agents have challenged hospitals in recent decades. Our aim was to investigate the circulation of target infectious agents using Geographic Information System (GIS) and spatial–temporal statistics to improve surveillance and control of healthcare-associated infection and of antimicrobial resistance (AMR), using *Klebsiella pneumoniae* complex as a model.

**Methods:**

A retrospective study carried out in a 450-bed federal, tertiary hospital, located in Rio de Janeiro. All isolates of *K. pneumoniae* complex from clinical and surveillance cultures of hospitalized patients between 2014 and 2016, identified by the use of Vitek-2 system (BioMérieux), were extracted from the hospital's microbiology laboratory database. A basic scaled map of the hospital’s physical structure was created in AutoCAD and converted to QGis software (version 2.18). Thereafter, bacteria according to resistance profiles and patients with carbapenem-resistant *K. pneumoniae* (CRKp) complex were georeferenced by intensive and nonintensive care wards. Space–time permutation probability scan tests were used for cluster signals detection.

**Results:**

Of the total 759 studied isolates, a significant increase in the resistance profile of *K. pneumoniae* complex was detected during the studied years. We also identified two space–time clusters affecting adult and paediatric patients harbouring CRKp complex on different floors, unnoticed by regular antimicrobial resistance surveillance.

**Conclusions:**

In-hospital GIS with space–time statistical analysis can be applied in hospitals. This spatial methodology has the potential to expand and facilitate early detection of hospital outbreaks and may become a new tool in combating AMR or hospital-acquired infection.

**Supplementary Information:**

The online version contains supplementary material available at 10.1186/s13756-021-00944-5.

## Background

The emergence and spread of antimicrobial resistance (AMR) in recent decades have challenged hospitals, especially by Gram-negative bacteria with multiple-drug resistance (MDR) mechanisms, as the development and access to the therapeutic options for treating infections caused by these agents are still limited. In addition, bacterial cross-transmission between patients and transfer of AMR genes between bacteria are increasingly more well recognised in hospitals [1–3]. Consequently, the incidence of healthcare-acquired MDR Gram-negative bacteria has increased dramatically over the last decades making AMR monitoring and containment critical.

Currently, *Klebsiella pneumoniae* is a good example of a hospital-acquired pathogen that often causes colonisation and infection, with high morbidity and mortality rates and costs [4–7]. The global expansion of hypervirulent and MDR clones of *K. pneumoniae* has been increasingly reported, especially of carbapenem-resistant strains [5, 8–14]. In the study hospital, we experienced an evolving epidemiology among *K. pneumoniae* with clonal PDR/XDR KPC-2-producing ST437 strains causing intractable infections between 2014 and 2015 [7]. These events led us to investigate a new approach to monitor AMR, since effective control requires a detailed understanding of how the dynamics of the agent’s circulation in the hospital occur.

Geographic information system (GIS) is a well-known system that aims to visualise, explore and model in a fast, direct, simple and interactive way, thus allowing mapping of events and enabling the analysis of spatial information in different degrees [15–17]. Although GIS is widely used in health [15–17], it has not been well explored for surveillance of infectious agents throughout hospitals [18].

Investigating the circulation of MDR bacteria, specifically using members of *K. pneumoniae* complex as a model, with spatiotemporal epidemiological techniques within a hospital unit, is a method that may identify critical areas and outbreaks, helping to improve surveillance and control of healthcare-associated infection and may prevent dissemination of AMR microorganisms. We hypothesised that this novel methodology would have sufficient accuracy to be performed indoors to facilitate the monitoring of any infectious agents and AMR profiles interactively. Therefore, our main goal was to demonstrate the feasibility and gains of conducting surveillance of a target infectious agent by using routine monitoring data to detect clusters through robust spatial epidemiological tools in a hospital.

## Methods

### Study design, setting and period

This retrospective study was carried out in a 450-bed federal tertiary hospital located in Rio de Janeiro. This research has been approved by the institutional review board with an informed consent waiver. We followed ORION statements in this study report [19].

All isolates of *K. pneumoniae* complex were investigated from the database of the hospital's microbiology laboratory*,* without repetition (only the first isolate was used whenever more than one isolate of *K. pneumoniae* complex was detected in the same biological sample collected on the same day), recovered from clinical and surveillance samples from patients hospitalised in intensive (ICU) and nonintensive care wards in the years of 2014 to 2016. We did not perform any exclusion criteria in the data collection phase, but only in the analysis phase of the study, therefore, only the patient’s first isolate with the specific phenotype was used to estimate incidence density rates and identify carrier status.

### Hospital physical structure and basic map construction

A basic longitudinal map with a scale of the hospital's physical structure was developed in AutoCAD (DWG archives) and converted to QGis software (version 2.18, Open Source Geospatial Foundation), providing the construction of the first version of the hospital’s GIS. All intensive (*n* = 6) and nonintensive (*n* = 73) care wards located on the 11th and 5th floors of the main and annex buildings, respectively, were mapped. Additional file 1: Table S1 shows the number of floors, units and beds of each building and Additional file 1: Fig. S1 shows their features mapped in QGis.

### Microbiological samples, bacterial identification and susceptibility testing

Clinical samples for microbiological exams were routinely collected by primary physicians as part of the investigation of infectious processes, guided by microbiological protocol implemented throughout the institution by the Hospital Infection Control Committee (HICC), during the study years. Rectal swabs for surveillance were collected weekly or every 15 days at all ICUs and systematically performed in nonintensive hospital areas according to the institutional HICC and microbiology laboratory protocol (Additional file 1: Table S2). Identification and antibiotic susceptibility testing of recovered strains were performed by conventional automated Vitek-2 system (BioMérieux, Marcy l′Etoile, France), including those from swabs. Etest and disk diffusion (Oxoid, Hampshire, UK) methods were used whenever indicated by the updated recommendation of the Clinical and Laboratory Standards Institute and European Committee on Antimicrobial Susceptibility Testing [20, 21]. Additional file 1: Table S3 shows the tested antibiotics. Screening for carbapenemase production was established by phenotypic tests with phenylboronic acid, EDTA and cloxacillin in a nonsystematic way by using carbapenemase inhibition test (CIT), as described elsewhere [22–24]. We classified the resistant profile of strains as non-MDR, MDR, possible extensively-drug (XDR) or pandrug (PDR) resistant, according to Magiorakos et al. [25]. Carbapenem-resistant *K. pneumoniae* (CRKp) complex isolates randomly preserved during this period and sent to Hospital Infection Research Laboratory of FIOCRUZ were also investigated to confirm microbial identification through classical methods [26] and for the search of carbapenemase genes (*bla*_KPC-2_, *bla*_NDM-1_ and *bla*_OXA-48-like_) using in-house multiplex polymerase chain reaction.

### Patient data

All inpatients with the detection of *K. pneumoniae* complex in any biological sample had the hospital records investigated for date, bed, ward and clinic of admission, date of discharge and transfer between beds. Patients were considered to be colonised or infected with carbapenem-resistant strains after the first detection of isolates with this resistance profile, for the remaining hospitalisation period and during readmissions.

### Georeferencing of bacteria and patients

The occurrences of members of *K. pneumoniae* complex with the susceptibility profile classified as above and also those detected as carbapenem resistant were entered manually into the QGis software and plotted on the map and georeferenced according to hospital ward and detection date. Likewise, patients harbouring CRKp complex were georeferenced for the duration of hospitalisation. In other words, we georeferenced different bacteria phenotypes and patients carrying a specific phenotype. When georeferenced bacteria phenotypes we consider all agents, with the exclusion criteria mentioned above. While when georeferenced patients carrying a specific phenotype, we considered only the first isolate with the specific phenotype (CRKp complex) by the period of hospitalisation. Thus, the circulation of patients carrying CRKp complex was investigated in space and time.

### Statistical analysis

A descriptive analysis was performed by using R statistical package (R Core Team, 2019) and a spatial statistical analysis was performed by using SaTScan program [27]. In all analysis *p* value ≤ 0.05 was considered significant for both sides of the curve.

#### General statistical analysis

Exploratory data analyses were performed in the study. We used non-parametric Mann–Whitney and Kruskal–Wallis tests for comparisons between groups, taking into account the total number of isolates and their susceptibility profiles, per month of detection or for the period of hospitalisation. Tests for equal proportions between groups were also used. We were not able to construct denominators (total patient-days) per ward of admission, but we used this information from the entire institution to estimate the incidence density of *K. pneumoniae* complex phenotypes during the study period. We estimate the monthly incidence density of target bacterial phenotype, considering only the first isolate of the patient with the respective phenotype per month, divided by the monthly total of patient-days normalised by 1000. In time series analysis, we used the approximate Cox–Stuart trend test, we performed seasonal and trend decomposition based on Loess (STL) and we examined anomalies [28].

#### Spatial statistical analysis

Spatial statistical analyses were performed in order to detect clusters of patients harbouring CRKp complex through the SaTScan program (available in www.satscan.org). Two sets of data analysis were done. One of them aimed to verify the distribution and spatial pattern in the occurrence of *K. pneumoniae* complex isolates with the specific phenotype (non-MDR, MDR, possible-XDR or -PDR and CRKp) recovered from patients hospitalised in the study period, regardless of whether clinical or surveillance material, and according to the date, month and year in which the isolate was detected. Another set of data analysis was based on patients with a specific phenotype (CRKp complex) counted monthly from the day of the first detection to the date of hospital discharge or death. The X (horizontal distance) and Z (floor height) coordinates refer to lateral and height distances (between floors) amidst the centroids of the wards. The neighbourhood (proximity matrix) was created based on the Euclidean distance [29], i.e., the horizontal and vertical distance in meters between pairs of wards, assuming that nearby wards can be contaminated by the use of common equipment, the circulation of health professionals and visitors. The transfer of clustered patients between wards was investigated using a flow map connecting the origin and destination wards using arrows, indicating colonisation or infection by CRKp complex based on microbiological results routinely collected.

As no population-at-risk data were analysed by wards, the expected number of positive samples for each ward was calculated, using only the total number of cases, considering the stability in the number of sample collections and hospitalisations per month. In other words, the proportion of all observed cases that occurred in each ward by the total number of cases expected during the month. The space–time permutation model was chosen in this case since it dismisses denominators since the absolute number of cases are compared [27].

The spatial scan statistic using a space–time permutation model was performed by overlapping circles, in which their domains represented the two-dimensional (X and Z) space of the hospital, and a third dimension represented time (month) to define the scan window. Thus, the number of monthly cases observed was counted and compared in and out of the circle, with the expected value being the average number of cases during the overall study period. The program marks the set of medical wards inside the circles with a statistically significant (*p* < 0.05, assuming a Poisson statistical distribution) difference between the observed and expected number of cases. Circles represent clusters of cases, and can thus comprise a finite number of neighbour wards within a period of several months. Once these circles are identified, secondary indicators are calculated, such as the maximum likelihood ratio, statistical significance of clusters, and relative risks (RR). The circles’ centre coordinate and circle radius (in meters) are also outputs from the program and were used to plot hot spots into the digital maps in GIS.

## Results

### Georeferenced *K. pneumoniae* complex and resistant phenotypes

Among the recovered information of a total of 759 K*. pneumoniae* complex isolates from 564 patients, 40% (*n* = 303* isolates*) was retrieved from ICU and 60% (*n* = 456) from other noncritical areas (Additional file 1: Algorithm 1). Additional file 1: Table S1 shows the number of isolates per clinic in the main and the annex buildings and Additional file 1: Fig. S2 shows the mapped distribution of the isolates during the 3-year period. Two-thirds (509/759) of the isolates were detected from clinical samples such as urine (46%, 232/509), blood (29%, 146/509), and respiratory tract samples (9%, 48/509). The ratio of isolates per patient was 1.3:1, and 77% (432/564), 17% (94/564) and 6% (35/564) of patients had, respectively, 1, 2 and ≥ 3 strains recovered from different samples during the study. Only 3% (19/564) of the patients had isolates detected in different samples from the same day. Different and progressively more resistant phenotypes were detected respectively in 36% (47/129) and 23% (29/129) of our patients with two or more isolates. Fifty-nine percent (17/29) of the patients with progressively more resistant phenotypes had the isolates detected in different months. Surveillance rectal swabs accounted for 33% (250/759) of isolates, with 74% of those recovered from ICUs (186/250). Resistance to any carbapenems (investigated in the entire hospital) was found in 76% (189/250) of rectal swab isolates and 24% (61/250) had carbapenem susceptible ESBL-positive phenotype (investigated mainly in paediatric wards).

In addition to the increase of *K. pneumoniae* complex detection between 2014 and 2016, corresponding to a similar proportion of less than 1% of the total number of microbiological exams performed in the hospital in 2014 (0.7%, 212/32, 560; 95% CI: 0.56–0.75%), 2015 (0.8%, 260/31, 364; 95% CI: 0.73–0.94%) and 2016 (0.9%, 287/33, 290; 95% CI: 0.77–0.97%), a significant increase (*p* < 0.001) of possible XDR/PDR strains was identified from 2014–2016 and shown by this spatial methodology (Additional file 2: Fig. S3). This was also verified by the monthly incidence density, per 1000 patient-days, of patients with *K. pneumoniae* complex phenotypes investigated in this study (Additional file 1: Fig. S4). Almost all possible XDR/PDR strains (99%, 164/166) displayed resistance to carbapenems.

### CRKp complex and time series analysis

Among CRKp complex (35%, 265/759), which corresponded to 56% (265/472) of the MDR strains recovered from 175 patients (1.5:1 isolates per patient ratio), in which 28% (*n* = 49) and 17% (*n* = 30) of the patients had two or more isolates detected, respectively, during the study period and at the same month. Only 7% (18/265) of isolates were detected in the first 48 h of admission. Therefore, 93% (247/265) of isolates were associated with hospitalisation. Resistance to meropenem was diagnosed in 99% (213/214) of tested isolates, with high-level resistance (minimum inhibitory concentration, MIC ≥ 16 μg/ml) in 88% (187/213). For imipenem tested isolates 61% (107/175) had MIC ≥ 16 μg/ml (Additional file 1: Fig. S5). One hundred percent of clinical tested CRKp complex strains (n = 38) had a positive screening for the production of carbapenemase. *bla*_KPC-2_, *bla*_OXA-48-like_ and *bla*_NDM-1_ were detected respectively in 83% (71/86), 7% (6/86) and 6% (5/86) of preserved CRKp complex from rectal swabs (*n* = 50) and clinical samples (*n* = 36), confirming 91% (78/86) with this mechanism of resistance.

In time-series analyses, regardless of biological material, whether clinical or surveillance, there was a significant increase (*p* value < 0.001) in the occurrence of patients harbouring CRKp complex (Additional file 1: Fig. S6). This was confirmed by STL decomposition, which also showed a seasonal increment in the second half of each year (Additional file 1: Fig. S7), irrespective of clinical or surveillance material and without the interference of detected anomalies (Additional file 1: Fig. S8).

### Georeferenced CRKp complex isolates

The increased in CRKp complex carriers is shown unequivocally and in a more specific way by the annual hospital GIS (Additional file 1: Fig. S9) with mapped occurrences by wards. In general, intensive- or nonintensive-care adult wards (258/265, 97%) had a significantly higher frequency of carbapenem-resistant strains (total number of CRKp complex isolates divided by the total number of *K. pneumoniae* complex detected, multiplied by 100) than paediatric wards of any kind (7/265, 3%) (*p* = 0.02) (Additional file 1: Fig. S9). The occurrences in paediatrics were predominantly of surveillance swabs (*n* = 6) with only one blood sample from Paediatric ICU. Maternity had 1 rectal swab only. CRKp complex was more prevalent in medical wards than in surgical or clinical-surgical ones (*p* ≤ 0.001). The occurrence of patients harbouring CRKp complex was higher in ICU than in non-ICU (*p* ≤ 0.001). Floors with ICUs (*p* ≤ 0.001), and wards with multiple beds when compared to single rooms (*p* ≤ 0.001), had higher occurrences of CRKp complex. The adult ICU (#100 on map) had a high and constant occurrence throughout the period, regardless of clinical or surveillance material. Investigation of space and time circulation of all patients carrying CRKp complex showed that although adult ICU was the most frequent site of first detection (Additional file 1: Fig. S10B), non-critical sectors also pressured this ICU with transfer of colonised/infected patients (Additional file 1: Fig. S10C).

### Space–time clusters of patients with CRKp complex infection/colonisation

**Fig. 1:**
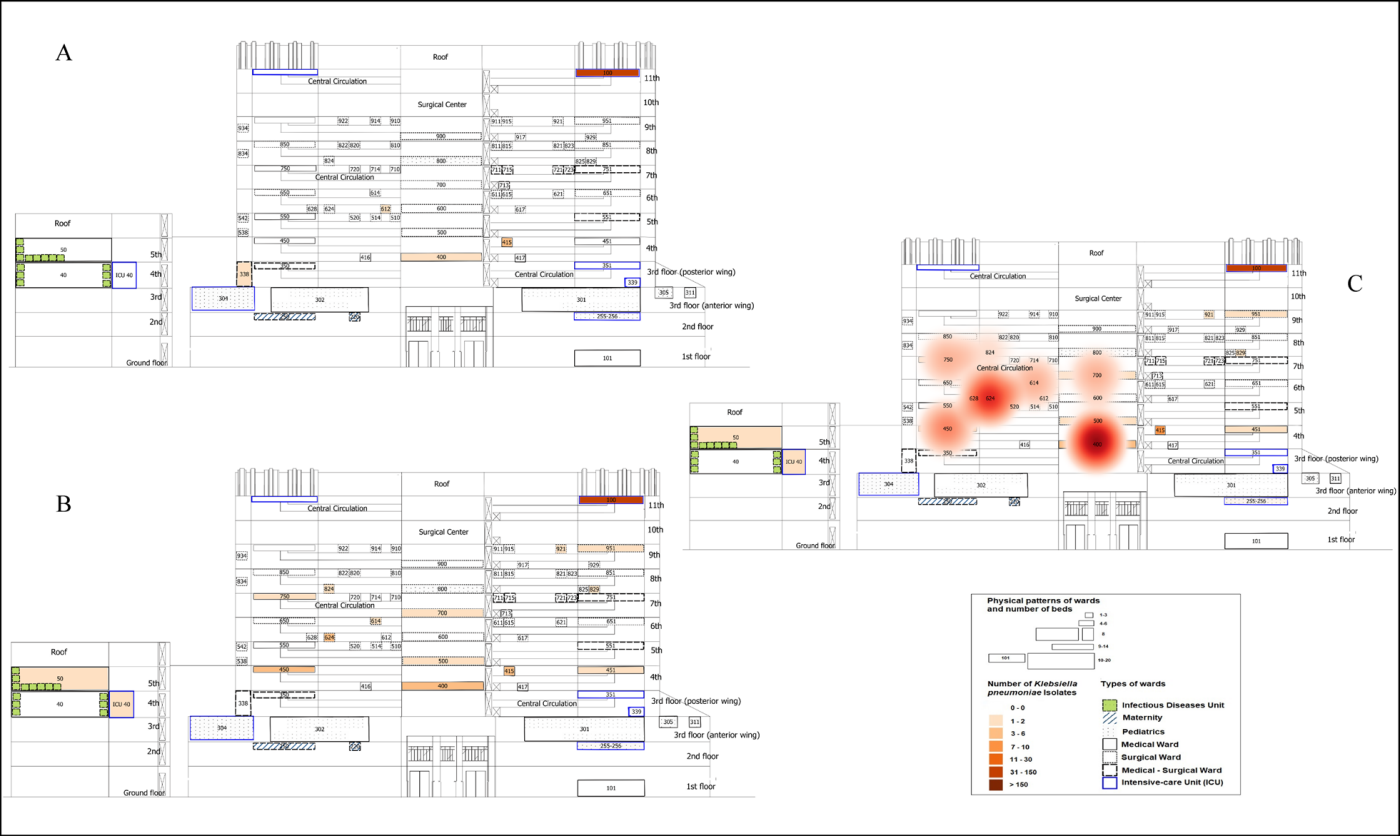
Cluster of patients harbouring carbapenem-resistant *Klebsiella pneumoniae* complex, tertiary federal hospital. **a** Pre-cluster period, 1st semester 2014; **b** Cluster period, 2nd semester 2014; **c** Cluster represented in heat map layer in red tones, the higher the colours tone the higher the occurrence, 2nd semester 2014 (affecting the wards of numbers 400, 450, 500, 614, 624, 700, 750, 824), *p* = 0.0016

**Fig. 2:**
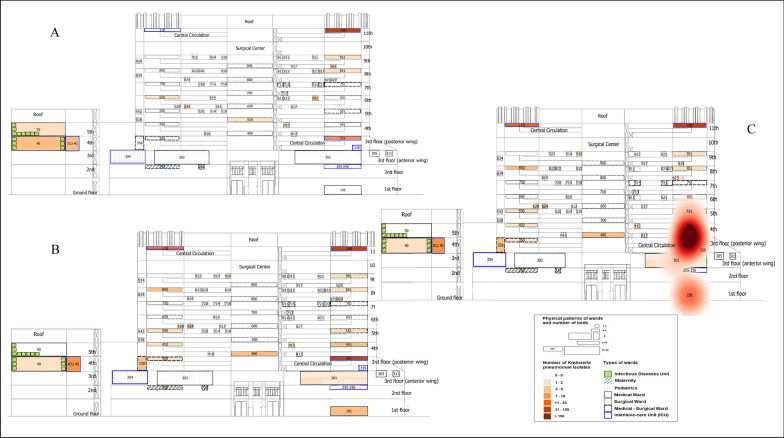
Cluster of patients harbouring carbapenem-resistant *Klebsiella pneumoniae* complex, tertiary federal hospital. **a** Pre-cluster period, January to September 2015; **b** Cluster period, 4th quarter 2015 to 1st semester 2016; **c** Cluster represented in heatmap layer in red tones, the higher the colours tone, the higher the occurrence, 4th quarter of 2015 and 1st semester of 2016 (affecting the wards of numbers 101, 301, 351, 451, 551), *p* = 0.004

The first detected cluster of patients with CRKp complex occurred in wards located on the central and left side of the main building, during the entire second half of 2014 (RR = 2.73, *p* = 0.0016) (Fig. 1). This cluster was affecting 8 adult patients representing 13.1 patient-months (the total number of bed-months that the affected patients contributed to the cluster) (Additional file 1: Fig. S11 and S12) or 394 patient-days (the total number of bed-days that the affected patients contributed to the cluster) of colonisation in the cluster area (clinical, clinical-surgical or surgical wards located on the 4th to 8th floor). A second cluster occurred on the right side of the central building also on different floors (Fig. 2), extending from October 2015 to mid-2016 (9 months duration) (RR = 1.91, *p* = 0.004). This cluster affected 14 adults and 1 child representing 28.8 patient-months (Additional file 1: Fig. S11 and S12) or 865 patient-days of colonisation in the cluster area (coronary ICU, internal medicine, general duty and a paediatric ward). The epidemiological and microbiological characteristics of patients involved in the first and second cluster are shown in Additional file 1: Table S4. None of these clusters was perceived by the regular AMR surveillance and control program performed daily by the HICC (Additional file 1: Table S5), although 87% (20/23) of clustered isolates represented hospital-associated infection or colonisation.

**Fig. 3:**
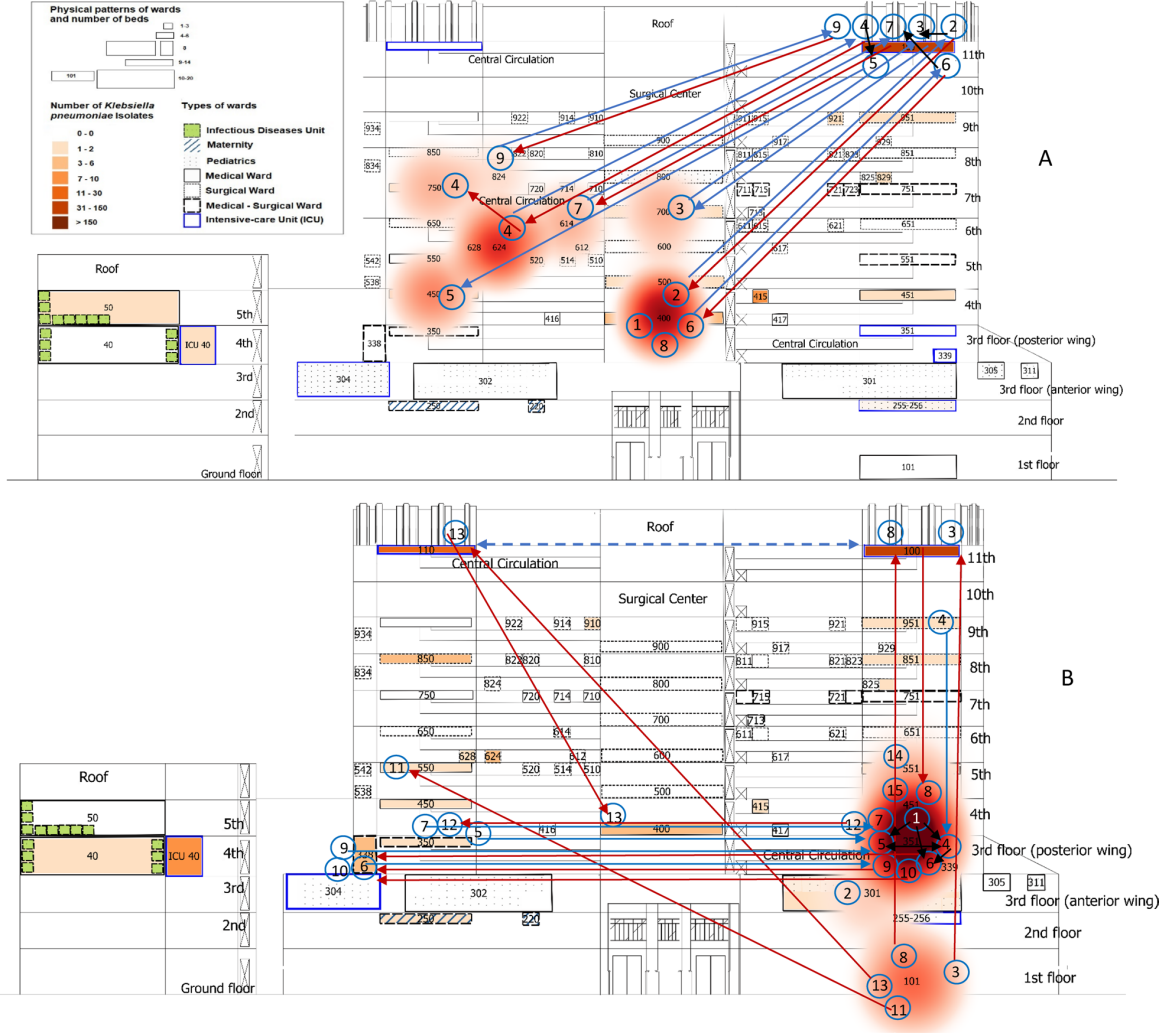
Patient flow maps during the first (**a**) and the second (**b**) clusters showing the transfers of clustered patients (arrows) between wards. The cluster is represented in red heat map layer with the occurrences of patients harbouring CRKp complex during the 2nd semester 2014 (first cluster) and the 4th quarter 2015 to 1st semester 2016 (second cluster). Patient numbers are in blue circles ordered by the date of the first detection, including the first detected case in the pre-cluster period of the first cluster (patient#1). The blue and red arrows represent the transfer of a clustered patient, respectively, before and after the first detection of CRKp complex isolate. The black arrow represents the opportunity for bacteria transmission between patients, considering the admission ward or unit and hospitalisation period after the first detection. While dashed blue arrow in (**b**) has the meaning to demonstrate that these units work together as the same ICU. Patients #1 and #8 in the first cluster and patients #2, #14 and #15 in the second group never moved to another ward and, among them, only patient #8 had the direct opportunity to acquire the bacteria from another clustered patient (patient #2) hospitalised in the same period at the same ward (#400)

Patient flow maps (Fig. 3) and Additional file 1: Fig. S12 show the epidemiological link between clustered patients and clearly demonstrated that the adult ICU (ward #100) was involved in both clusters, but in the second cluster, the coronary ICU (ward #351) was the hot spot affecting neighbouring wards. Only three strains (patients #3, #8 and #13) of the second cluster had carbapenemase genes investigated and all were KPC-2 producing and negative for NDM-1 and OXA-48 genes. These patients have never met physically during the hospitalisation period, although they were admitted to the same wards (Fig. 3, Additional file 1: Fig. S12). Seven out of nine patients (78%) in the first cluster and 27% (4/15) in the second cluster had an opportunity for bacteria transmission among them. Figure 3 also shows the patient’s status of colonisation/infection by CRKp complex during transfers between intensive and non-intensive wards.

## Discussion

The use of in-hospital GIS associated with the spatial statistics for endemic level monitoring and cluster detection of patients harbouring AMR bacteria is feasible. As far as we know, this is the first study to develop a surveillance methodology using GIS and spatial statistics to investigate and monitor target microorganisms causing infection/colonisation in patients from all sectors of the hospital for 3 consecutive years.

In spite of the literature recommending the use of GIS [18, 30], and spatial statistics for data analysis in hospitals and health units for almost two decades [17, 31], none of the studies used both tools for AMR surveillance and the number of in-hospital studies remains very incipient [17, 18, 30, 31]. Several sites and applications nowadays use GIS to gather and monitor infectious disease data worldwide (https://www.healthmap.org/en/; https://omictools.com/biocaster-tool; https://www.istm.org/geosentinel). These are bio-surveillance systems that capture, organise and analyse public health information that can be accessed by anyone on the internet, but none of them has applicability to in-hospital epidemiology. A new version of WHONET 2019 (http://www.whonet.org/index.html) uses Martin Kulldorf's trademark SaTScan™ to detect space–time clusters, including MDR bacteria in hospitals. The site reports the same statistical method used in our study in a 5-year project, but with no mention of in-hospital GIS.

The occurrence of MDR Gram-negative microorganisms in hospitals increases geometrically and surveillance manuals are regularly renewed [32–35], while methodologies for mapping events and detecting clusters in hospitals need to progress at the same pace [13]. The spread of *K. pneumoniae* producing KPC carbapenemases has become an unparalleled public health crisis [7–11, 14]. In the studied hospital, the impact of evolving epidemiology with a global dimension was not different with an important increase in the resistance profiles of *K. pneumoniae* complex over the years [7].

Clusters of patients with CRKp complex in different hospital areas and floors, where most workflows are generally differentiated, would probably be overlooked by the surveillance methodologies currently used to control hospital-acquired infections. In the literature, we found no publications mentioning similar bacterial outbreak affecting adult and paediatric units located on different hospital floors. This partly explains why these were not noticed by the hospital surveillance system that used methodology without GIS and spatial statistics. Moreover, clusters affecting non-critical areas with lower frequencies were likely disregarded due to the higher occurrence in ICUs, where the higher-level resistance profile (concomitant resistance to polymyxin) probably attracted more attention from our HICC [7]. The use of both technologies offered the advantage of monitoring a large number of data and different resistance patterns in hospital clinical areas with different endemic levels.

In this study, Additional file 1: Fig. S11 and S12 represent the traditional methodology for mapping occurrences without GIS and spatial statistics, while Figs. 1, 2 and 3 and Additional file 1: Figures S2, S9, S10 and Additional file 2: Figure S3 represent the new method for mapping the occurrences. The visual aspect of the new method contributes to understanding the complexity of in-hospital surveillance, whilst the detection of spatial–temporal clusters affecting diverse clinics on dissimilar floors, during the period of months, is likely only possible by using the space–time clustering method.

The agglomeration in time and space and flow of patients harbouring CRKp complex indicates the need to investigate the clonal aspects of isolates and the sources of dissemination. Therefore, molecular genetics of the microorganism is a necessary step for precise discrimination of *K. pneumoniae* from related phylogroups and genotyping [36]. There are several potential sources of transmission of *K. pneumoniae* complex that needs to be further explored in the hospital [5]. Radiology, operating rooms, support services such as hospital hygiene, laboratory, nutrition and even consulting professionals serving different areas of adults and paediatrics could serve as vehicles or sources of transmission in different floors and units. Silent colonisation is useful to explain some of our results [13, 37]. This becomes apparent when the resistant organism was detected in other clustered patients who never met physically but were hospitalised in the same wards.

The clusters last a few months in diverse neighbouring wards, indicating a continuous and widespread circulation of resistant bacteria between intensive and nonintensive care areas. The possibility of infection spread from critical wards to nearby health units throughout months is a clue to preventive measures [13]. Although real-time spatial monitoring of AMR can provide early warnings for infection control from its origin, potential carriers (equipment, health professionals, etc.) and transmission routes, bacterial genotyping is required for investigating in-hospital bacterial transmission [13]. The lack of genetic analysis of isolates is an important limitation in this study, especially for confirming outbreaks.

The addition of the use of hospital GIS and spatial statistics to other surveillance measures already used in hospitals, such as previous antimicrobial consumption, can be an important milestone for renewing the monitoring and control of diseases that kill thousands worldwide each year. The management of several infections and activities that take place within a hospital can benefit from this GIS methodology to ensure the safety of patients and healthcare professionals. Georeferencing patients, staff, visitors, or even hand hygiene locations and hours between or in contact with inpatients, among other factors that involve this issue, would help to understand and control the main sources and routes of transmission of microorganisms in hospitals [18, 30]. Further studies of this nature would improve the knowledge about the dynamics of microorganisms and thus contribute to the construction or remodelling in order to better meet workflows and prevent the circulation of infectious agents and the risk of transmission. This interactive and visual approach, specifically demonstrating which room, ward, corridor, floor, not just the clinic, and whenever events begin, would be performed prospectively in real-time and in 3D to proactively impact the health professionals’ adherence to infection prevention and control in the future.

Although challenging, the methodologies using georeferencing software are on the internet. QGis software is free to download (https://qgis.org/en/site/forusers/download.html). Hospitals often have architects who support the construction and renovation of physical facilities. The application of spatial statistics would require skilled professionals, but more and more technologies that enable interactive learning or the use of this tool are becoming available on the internet (http://www.whonet.org), and hospitals could incorporate specialised personnel. Additionally, integrated real-time GIS components have been used for real-time surveillance [38]. Similarly, the microbiology laboratory system can be integrated with QGis and R, since both statistical packages allow for many spatial analyses in an automated mode [39].

Since this is a retrospective study, several limitations are expected. First, our intention was to demonstrate a new approach to detect clusters, which can be simply defined as an aggregation of cases grouped in place and time [40]. We did not aim to investigate outbreaks [40], because of the retrospective nature of the study. We cannot provide the analysis of infectious rates because we worked only with the hospital’s microbiology laboratory database. We investigated real-life data of *K. pneumoniae* complex isolates recovered from all clinical and surveillance material and excluded only isolates from the same biological sample collected on the same day. We did this at the beginning to make sure we would not introduce any bias into the study, since all laboratory results are important for spatial and temporal monitoring of agents’ AMR phenotypes.

Considerable variation in AMR data analysis still present and some of recommendations were made for reporting [41], but not for real-life surveillance as our purpose. Since we monitored different phenotypes of *K. pneumoniae*, we cannot include the first isolate from each patient only. This is especially important because different phenotypes can circulate concomitantly in the same patient, in blood and rectal swabs for example. In addition, the emergence of resistance during hospitalization in a given species in the same patient can only be suspected by following all isolates and phenotypes [7]. We chose to use in this study the same and minimum data that the HICC works initially and daily to monitor bacterial agents. Additional file 1: Figure S2 and Additional file 2: Figure S3 represent georeferenced bacteria phenotypes considering all agents included, so one limitation of this approach is that we cannot counted each isolation as a different process of infection or colonization as a prior convention [42, 43]. While Figs. 1, 2 and 3 and Additional file 1: Figures S9 and S10 represent georeferenced patients considering the first isolate with the specific phenotype by the period of hospitalisation. Nevertheless, we probably investigated a smaller tip of the iceberg; sample collection was performed during the routine investigation of infectious processes in hospitalised patients, and carbapenem-resistant Enterobacteriaceae surveillance performed differently in critical and non-critical units. Thus, the results do not represent the actual occurrence.

The seasonal increase in CRKp complex carriers detected in time series analysis also deserves further investigation and may pose an additional challenge with regard to control outbreaks [11]. A multivariate analysis considering other variables such as length of hospital stay, previous use of antimicrobials and age would be important to better estimate rates. In addition, our data point to a statistical analysis of the longitudinal study considering a nonlinear multivariate regression, based on recent literature [44], with different levels of correlation among the same or different patients, in the same or in different wards.

Although colonised/infected patients are the main reservoirs and sources of bacterial circulation, health-care workers also participate in the dynamics of infectious agents in hospitals [34]. The transmission routes and contact network between clusters can also be investigated by space–time scan statistics [45]. The proximity matrix can be improved in the future, using data on the flow of patients, health professionals and medical materials. The 1-month timescale was designed to mimic what is normally reported in-hospital surveillance, but a more refined scale will be necessary in real life [45].

Using the space–time permutation model without the use of denominators, such as total patient-days or total admissions, considering the stability in the number of samples collected and hospitalisations (data not shown), may facilitate and improve resistance surveillance locally in hospitals, especially in low-income countries, as the construction of denominators by infirmaries requires greater robustness of information, which is not always feasible. Although this is a limitation to compare rates between institutions, it is also a strong point, as it simplifies the monitoring of AMR, facilitating early detection in a hospital. Space–time permutation scanning statistic is a methodology designed for the early detection of events that uses only case numbers, with no need for population-at-risk data [27, 46]. But spatial statistics can be used with denominators improving comparability among institutions.

Nevertheless, we consider that none of these limitations interfered with our purpose to demonstrate a different approach for cluster detection with the substantial amount of data that HICC usually works with. The GIS methodology was designed to attend the real-life monitoring and can be applied using different AMR monitoring criteria. Our observation is that the amount of microbiological data, with the variety of target AMR phenotypes in hospitals, requires automated methods using spatial statistics for cluster detection. New and traditional methodologies for hospital surveillance are likely complementary, but more studies are needed to compare the benefit of each approach, the reliability of the mathematical model chosen and how it would do better for surveillance of AMR or any infectious agent in hospital. Finally, but not least, the rates found, although underestimated, are useful to demonstrate the importance of the theme in the hospital.

## Conclusions

The spatial methodology can be applied in hospital; it was useful for detecting clusters and has the potential to facilitate the early recognition of hospital outbreaks, even in different wards and floors. Any healthcare-associated infection could likely benefit from this methodology, including non-border resistant bacteria. Spatial statistical analysis of georeferenced microorganism in hospitals may become a new instrument for health care institutions in the fight against target infectious agents and AMR.

## Supplementary Information

**Additional file 1: Table S1.** Distribution of wards and beds, and the number of *Klebsiella pneumoniae* complex isolates per type of ward, unit and floor in the Main and Annex buildings. **Fig. S1.** Thematic hospital map in QGis format. **Table S2.** Rectal swab protocol for surveillance of antimicrobial resistant Enterobacteriaceae. **Table S3.** Categories and antimicrobial agents used for susceptibility testing on *K. pneumoniae* complex. **Algorithm 1.** *K. pneumoniae* complex isolates included and excluded according to the hospital sectors of detection and the reason for exclusion. **Fig. S2.** Mapped distribution of *K. pneumoniae* complex isolates. **Fig. S4.** Monthly incidence density of patients infected/colonised by *K. pneumoniae* complex per 1000 patient-days according to respective phenotypes. **Fig. S5.** Minimal inhibitory concentration (MIC) of meropenem and imipenem among carbapenem-resistant *K. pneumoniae* (CRKp) complex recovered from inpatients. **Fig. S6.** Time series analysis of patients harbouring CRKp complex adjusted by number of microbiological exams performed monthly. **Fig. S7.** Seasonal Trend decomposition using LOESS (STL) - Time series analysis of patients harbouring CRKp complex. **Fig. S8.** Seasonal Trend decomposition for data anomaly taking into the account time series data of patients harbouring CRKp complex. **Fig. S9.** Pattern of annual distribution of patients harbouring CRKp complex. **Fig. S10.** Space and time circulation of all patients carrying CRKp complex by ward of admission; before, at the time and after the detection of CRKp complex colonisation or infection. **Fig. S11.** Number of patients infected or colonised by CRKp complex by clustered wards and month of hospitalization, during the first and the second pre-cluster, cluster and post-cluster period. **Table S4.** Epidemiological and microbiological characteristics and outcome of patients involved in the first and second cluster. **Fig. S12.** Epidemiological link between patients colonised or infected by CRKp complex during the first cluster and the second cluster.

**Additional file 2: Fig S3.** Thematic hospital maps of annual detection of *K. pneumoniae* complex according to the antimicrobial susceptibility profiles and regardless of clinical or surveillance samples.

## Data Availability

The datasets used and/or analysed during the current study are available from the corresponding author on reasonable request.

## Abbreviations

AMR: Antimicrobial resistance
CRKp: Carbapenem-resistant *K. pneumonia*
GIS: Geographic Information System
HICC: Hospital Infection Control Committee
ICU: Intensive care units
KPC-2: *K. pneumoniae* Carbapenemase 2
MIC: Minimum Inhibitory Concentration
MDR: Multidrug resistant
NDM-1: New Delhi metallo-beta-lactamase 1
OXA-48: Oxacillinase-48-like carbapenemases
ORION: Outbreak Reports and Intervention Studies of Nosocomial infection
PDR: Pandrug resistant
QGis: Quantum GIS
RR: Risk relative
STL: Seasonal and Trend decomposition based on Loess
XDR: Extensively drug resistant
