# Supplementary material for: Geographical information system and spatial–temporal statistics for monitoring infectious agents in hospital: a model using Klebsiella pneumoniae complex
Source: Antimicrob Resist Infect Control. 2021 Jun 16;10:92. doi: 10.1186/s13756-021-00944-5 (PMC8207788; doi:10.1186/s13756-021-00944-5)
Supplement: Supplementary file 1 — Additional file 1: Table S1. Distribution of wards and beds, and the number of Klebsiella pneumoniae complex isolates per type of ward, unit and floor in the Main and Annex buildings. Fig. S1. Thematic hospital map in QGis format. Table S2. Rectal swab protocol for surveillance of antimicrobial resistant Enterobacteriaceae. Table S3. Categories and antimicrobial agents used for susceptibility testing on K. pneumoniae complex. Algorithm 1. K. pneumoniae complex isolates included and excluded according to the hospital sectors of detection and the reason for exclusion. Fig. S2. Mapped distribution of K. pneumoniae complex isolates. Fig. S4. Monthly incidence density of patients infected/colonised by K. pneumoniae complex per 1000 patient-days according to respective phenotypes. Fig. S5. Minimal inhibitory concentration (MIC) of meropenem and imipenem among carbapenem-resistant K. pneumoniae (CRKp) complex recovered from inpatients. Fig. S6. Time series analysis of patients harbouring CRKp complex adjusted by number of microbiological exams performed monthly. Fig. S7. Seasonal Trend decomposition using LOESS (STL) - Time series analysis of patients harbouring CRKp complex. Fig. S8. Seasonal Trend decomposition for data anomaly taking into the account time series data of patients harbouring CRKp complex. Fig. S9. Pattern of annual distribution of patients harbouring CRKp complex. Fig. S10. Space and time circulation of all patients carrying CRKp complex by ward of admission; before, at the time and after the detection of CRKp complex colonisation or infection. Fig. S11. Number of patients infected or colonised by CRKp complex by clustered wards and month of hospitalization, during the first and the second pre-cluster, cluster and post-cluster period. Table S4. Epidemiological and microbiological characteristics and outcome of patients involved in the first and second cluster. Fig. S12. Epidemiological link between patients colonised or infected by CRKp complex during the [file 13756_2021_944_MOESM1_ESM.docx]

**Additional File 1**

**Table S1.** Distribution of wards and beds, and the number of *Klebsiella pneumoniae* complex isolates per type of ward, unit and floor in the Main and Annex buildings, tertiary federal hospital, Rio de Janeiro, 2014 to 2016

| **MAIN BUILDING** | | | |  |
| --- | --- | --- | --- | --- |
| **FLOOR** | **UNIT** | **WARD** | **# of BED** | **# of Isolates/per type of Ward** |
| **1st** | GENERAL DUTY | 101^a^ | 6 - 20 | 22 |
| **2nd** | MATERNITY | 220^c^ | 2 | 10 |
|  |  | 250^c^ | 14 |  |
|  | NEONATAL ICU | 255^d^ | 8 | 13 |
|  | NEONATAL IMCU | 256^d^ | 12 | 21 |
| **3rd (anterior wing)** | PAEDIATRICS | 301ª | 17 | 71 |
|  |  | 302ª | 10 |  |
|  |  | 305ª | 4 |  |
|  |  | 311ª | 1 |  |
|  | PEDIATRIC ICU | 304 | 8 | 32 |
| **3rd (posterior wing)** | CARDIOLOGY | 338^c^ | 8 | 25 |
|  |  | 339^c^ | 5 |  |
|  |  | 350^c^ | 12 |  |
|  | CORONARY UNIT | 351 | 12 | 45 |
| **4th** | INTERNAL MEDICINE | 400ª | 14 | 110 |
|  |  | 416ª | 1 |  |
|  |  | 417ª | 1 |  |
|  |  | 450ª | 14 |  |
|  |  | 451ª | 14 |  |
|  |  | 415ª^,e^ | 4 |  |
| **5th** | PROCTOLOGY | 500^b^ | 16 | 13 |
|  | OTORHINOLARYNGOLOGY | 510^b^ | 3 | 5 |
|  |  | 514^b^ | 3 |  |
|  |  | 520^b^ | 3 |  |
|  |  | 538^b^ | 3 |  |
|  |  | 542^b^ | 3 |  |
|  | INTERNAL MEDICINE | 550ª | 14 | 8 |
|  |  | 551^c^ | 14 |  |
| **6th** | ORTHOPEDICS | 600^b^ | 10 | 7 |
|  |  | 611^b^ | 1 |  |
|  |  | 615^b^ | 1 |  |
|  |  | 621^b^ | 1 |  |
|  |  | 617^b^ | 1 |  |
|  |  | 651^b^ | 14 |  |
|  | UROLOGY | 612^b^ | 6 | 14 |
|  |  | 614^b^ | 2 |  |
|  |  | 624^b^ | 1 |  |
|  |  | 628^b^ | 1 |  |
|  |  | 650^b^ | 14 |  |
| **7th** | NEUROSURGERY | 700^b^ | 11 | 7 |
|  | NEUROLOGY | 710ª | 2 | 25 |
|  |  | 750ª | 10 |  |
|  |  | 714ª | 2 |  |
|  |  | 720ª | 2 |  |
|  | GINECOLOGY | 713^c^ | 3 | 12 |
|  |  | 711^c^ | 2 |  |
|  |  | 715^c^ | 3 |  |
|  |  | 721^c^ | 3 |  |
|  |  | 723^c^ | 2 |  |
|  |  | 751^c^ | 14 |  |
| **8th** | PAEDIATRIC SURGERY | 800^b^ | 12 | 4 |
|  | GENERAL SURGERY | 810^b^ | 2 | 58 |
|  |  | 811^b^ | 2 |  |
|  |  | 815^b^ | 2 |  |
|  |  | 820^b^ | 2 |  |
|  |  | 821^b^ | 3 |  |
|  |  | 822^b^ | 2 |  |
|  |  | 823^b^ | 2 |  |
|  |  | 824^b^ | 2 |  |
|  |  | 825^b^ | 1 |  |
|  |  | 829^b^ | 1 |  |
|  |  | 850^b^ | 14 |  |
|  |  | 851^b^ | 14 |  |
|  |  | 834^b,e^ | 6 |  |
| **9th** | THORACIC SURGERY | 900^b,f^ | 14 | 9 |
|  |  | 910^b^ | 3 |  |
|  |  | 922^b^ | 2 |  |
|  |  | 914^b^ | 3 |  |
|  | VASCULAR SURGERY | 915^b^ | 2 | 12 |
|  |  | 917^b^ | 1 |  |
|  |  | 921^b^ | 2 |  |
|  |  | 911^b^ | 2 |  |
|  |  | 951^b^ | 14 |  |
|  |  | 929^b^ | 1 |  |
|  | PLASTIC SURGERY | 934^b^ | 6 | 4 |
| **10th** | SURGICAL CENTER | - | - |  |
| **11st** | ADULT ICU | 100^g^ | 10 | 159 |
|  | POSTOPERATIVE UNIT | 110^g^ | 9 | 27 |
| **ANNEX IV BUILDING** | | | |  |
| **FLOOR** | **UNIT** | **WARD** | **# of BED** |  |
| **4th** | INFECTIOUS DISEASES II | 40ª | 10 | 9 |
|  | INFECTIOUS DISEASES ICU | ICU40 | 4 | 17 |
| **5th** | INFECTIOUS DISEASES I | 50ª | 13 | 20 |

Note. ICU, Intensive care unit; IMCU, Intermediate care unit;

^a^ Medical ward; ^b^ Surgical ward; ^c^ Medical and surgical ward; ^d^ Occupying the same physical area;

^e^ Act as Intermediate Care Unit; ^f^ From October 2015 to December 2016 this area was occupied by Neonatal ICU and IMCU; ^g^ They work together as the same ICU


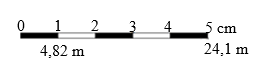

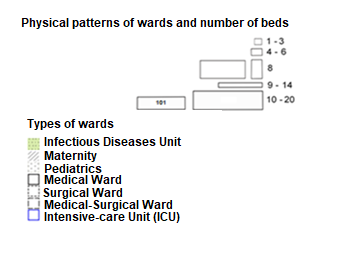

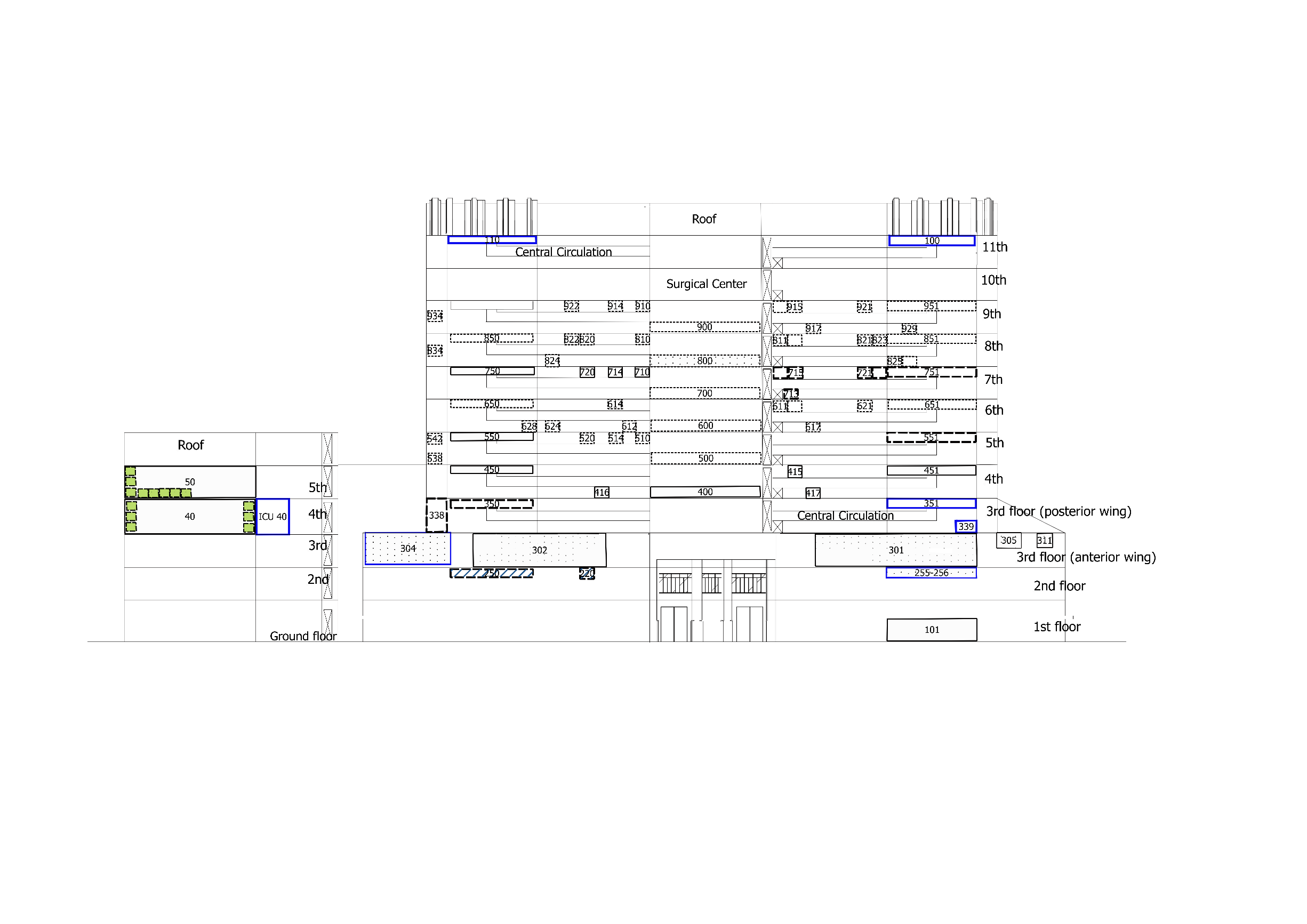


Right Wing

Left Wing

Footbridge

Central Wing

**MAIN BUILDING**

**ANNEX BUILDING IV**

**Fig. S1**. Thematic map in QGis format (version 2.18, Open-Source Geospatial Foundation), tertiary federal hospital, Rio de Janeiro, 2014 to 2016. The ward number positioned in the center of its respective physical area

**Table S2.** Rectal swab protocol for surveillance of antimicrobial resistant Enterobacteriaceae, Hospital Infection Control Committee and Microbiology Laboratory, tertiary federal hospital, 2014 to 2016

| **Target population** | |
| --- | --- |
| ICU patients, regardless of length of stay; | |
| Patients transferred from other hospitals, where they stayed for more than 12 hours; | |
| Patients transferred from other wards, where they stayed for more than 12 hours and/or underwent invasive procedures; | |
| Patients with vascular and pressure ulcers, and open surgical wounds; | |
| Patients hospitalised in the last 6 months prior to the current hospitalisation; | |
| Patients transferred from home care or outpatient parenteral treatment. | |
| **Rectal swab collection procedure^a^** |  |
| Always on admission of patients included in the definition of target population; |  |
| Weekly or biweekly in all ICU patients. |  |

^a^Rectal swabs were plated on commercially prepared CHROMagar medium (CHROMagar, France) supplemented with meropenem for the detection of carbapenem-resistant Enterobacteriaceae. In pediatric units, rectal swabs were also inoculated onto MacConkey agar plates (Oxoid, Lawrence, USA) for the detection of extended-spectrum beta-lactamase (ESBL)-producing Enterobacteriaceae.

**Table S3**. Categories and antimicrobial agents used for susceptibility testing on *K. pneumoniae* complex, tertiary federal hospital, 2014 to 2016

| **Antimicrobial category** | **Antimicrobial agent** |
| --- | --- |
| Aminoglycosides | Gentamicin |
|  | Tobramycin |
|  | Amikacin |
| Anti-MRSA cephalosporins | Ceftaroline |
| Antipseudomonal penicillins | Ticarcillin-clavulanic acid |
| + β lactamase inhibitors | Piperacillin-tazobactam |
| Carbapenems^a^ | Ertapenem |
|  | Imipenem |
|  | Meropenem |
| 1st and 2nd generation cephalosporins | Cefazolin |
|  | Cefuroxime |
| 3rd and 4th generation cephalosporins | Cefotaxime or ceftriaxone |
|  | Ceftazidime  Cefepime |
| Cephamycins | Cefoxitin |
| Fluoroquinolones | Ciprofloxacin |
| Folate pathway inhibitors | Trimethoprim-sulphamethoxazole |
| Glycylcyclines | Tigecycline |
| Penicillins | Ampicillin^b^ |
| Penicillins + β lactamase inhibitors | Amoxicillin-clavulanic acid |
|  | Ampicillin-sulbactam |
| Polymyxins | Colistin |

^a^ Carbapenem resistance was defined as *K. pneumoniae* complex isolates with nonsusceptibility to any of the following carbapenems: meropenem, imipenem, or ertapenem, as outlined by the Clinical and Laboratory Standards Institute (1); ^b^ Intrinsically resistant.

Incompleted data: 162 isolates

Postoperative unit under rebuilding in 2014: 10 isolates

Nonintensive care units: 20 isolates (4%, 20/476)

Intensive care units: 64 isolates (17%, 64/367)

84 isolates with detection clinic information

Excluded:

172 isolates (18%)

931 *K. pneumoniae* complex isolates, 2014 to 2016

**Included:**

**759 isolates** (82%) **from 564 patients**

**Intensive care units: 303 isolates** (40%)

**Nonintensive care units: 456 isolates** (60%)

**Algorithm 1**. *K. pneumoniae* complex isolates, without repletion, included and excluded according to the hospital sectors of detection and the reason for exclusion, tertiary federal hospital, 2014 to 2016.


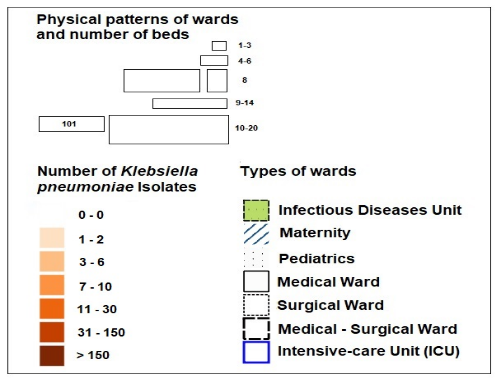

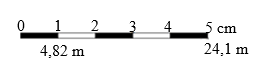

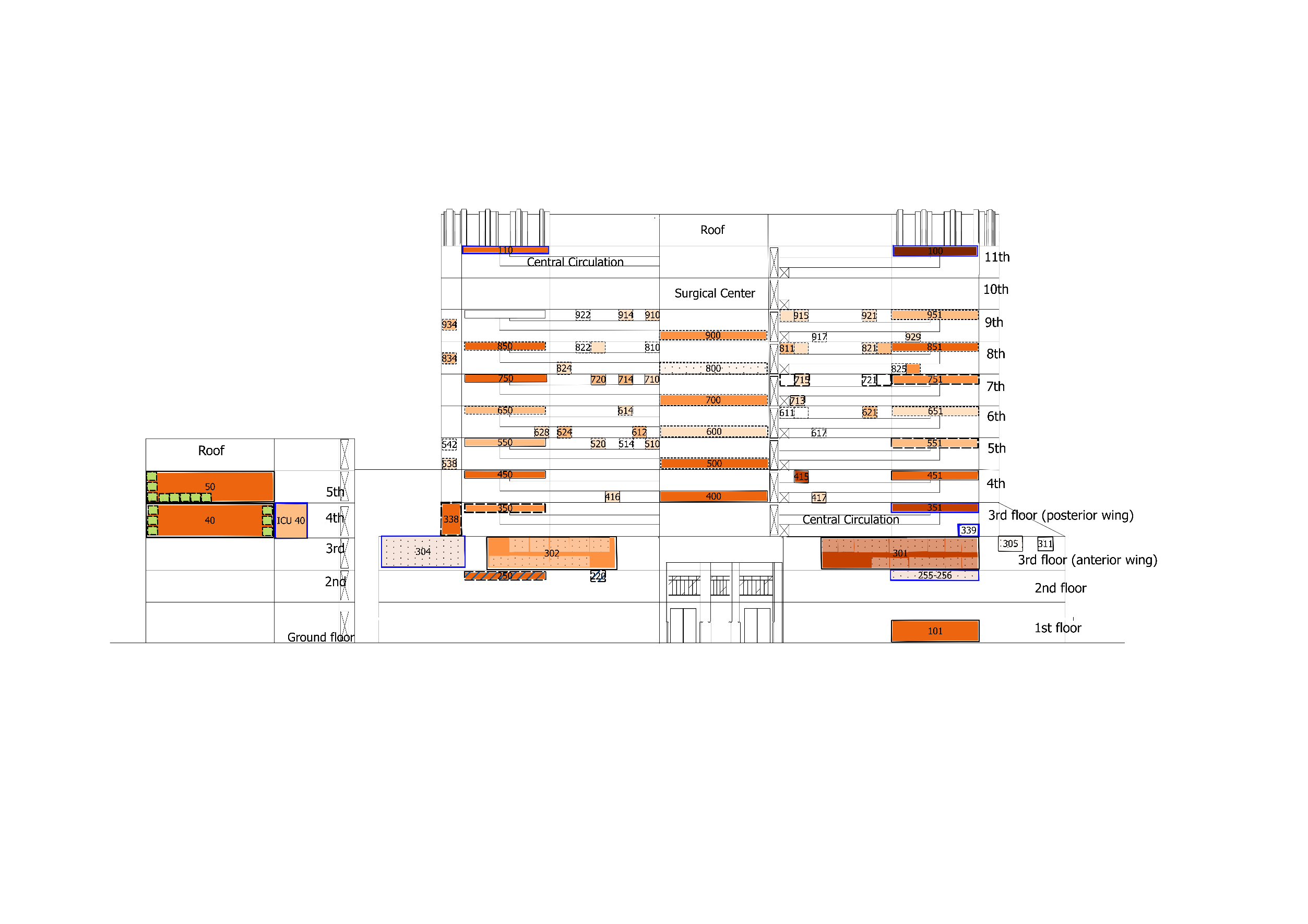


Footbridge

Right Wing

Central Wing

Left Wing

**ANNEX BUILDING IV**

**MAIN BUILDING**

**Fig. S2.** Distribution of *K. pneumoniae* complex (n = 759 isolates), regardless of clinical or surveillance samples and the antimicrobial susceptibility profile, tertiary federal hospital, 2014 to 2016. The ward number positioned in the center of its respective physical area

C

A

B

**Fig. S4.** Monthly incidence density of patients infected/colonised by *K. pneumoniae* complex per 1000 patient-days according to respective phenotypes (2), tertiary federal hospital, 2014 to 2016. **A**: regardless of clinical or surveillance material; **B**: clinical material only. MDR, Multidrug-resistant; XDR, Extensively-drug resistant; PDR, Pandrug resistant; CR, carbapenem-resistant

**Fig. S5**. Minimal inhibitory concentration (MIC) of meropenem (n = 213 isolates) and imipenem (n = 175 isolates) among carbapenem-resistant *K. pneumoniae* (CRKp) complex recovered from inpatients, regardless of clinical or surveillance samples, tertiary federal hospital, 2014 to 2016


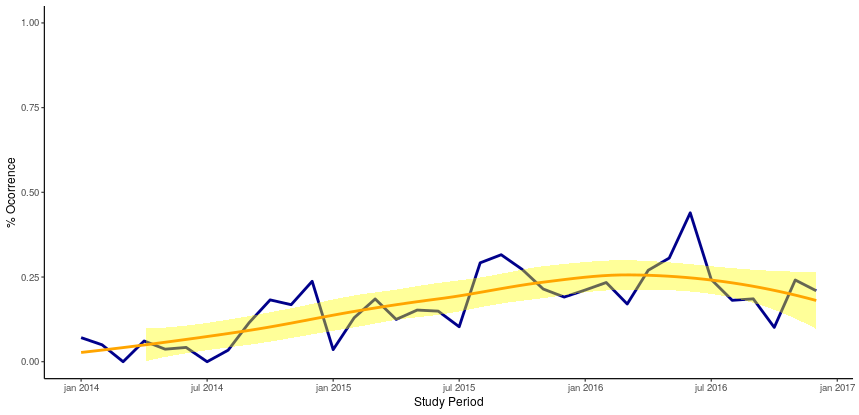

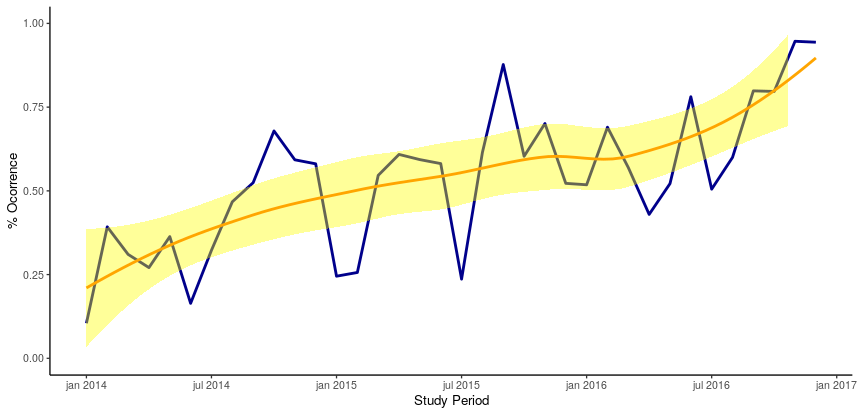


B

A

A

p < 0,001

**Fig. S6.** Time series analysis of patients harbouring CRKp complex adjusted by number of microbiological exams performed monthly, tertiary federal hospital, 2014 to 2016. A: regardless of clinical or surveillance material (n = 518 patients, 97,214 exams, p <0.001); B: clinical material only (n = 157 patients, 95,156 exams, p <0.0001). Approximate Cox–Stuart trend test (3).


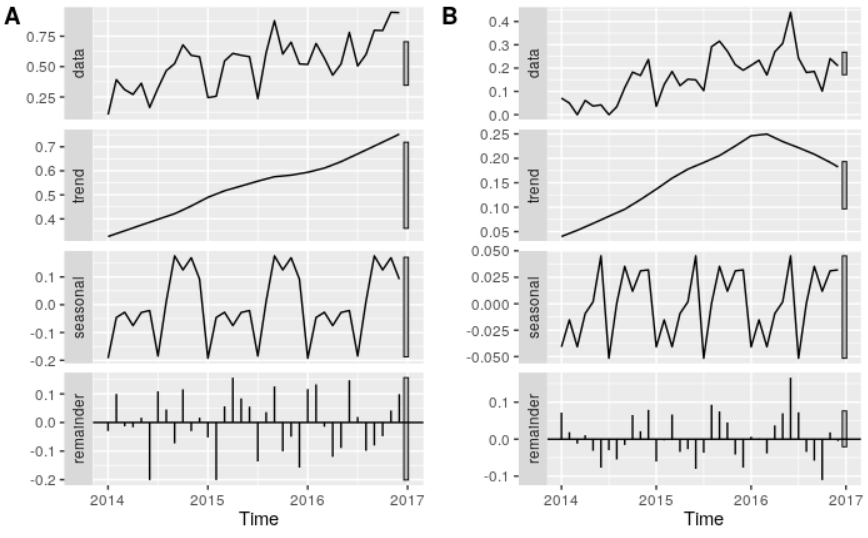


**Fig. S7.** Seasonal Trend decomposition using LOESS (STL) - Time series analysis of patients harbouring CRKp complex adjusted by the number of microbiological exams performed monthly, tertiary federal hospital, 2014 to 2016. A: regardless of clinical or surveillance material; B: clinical material only.

**Fig. S8.** Seasonal Trend decomposition for data anomaly taking into the account time series data of patients harbouring CRKp complex adjusted by the number of microbiological exams performed monthly, tertiary federal hospital, 2014 to 2016. A: regardless of clinical or surveillance material; B: clinical material only


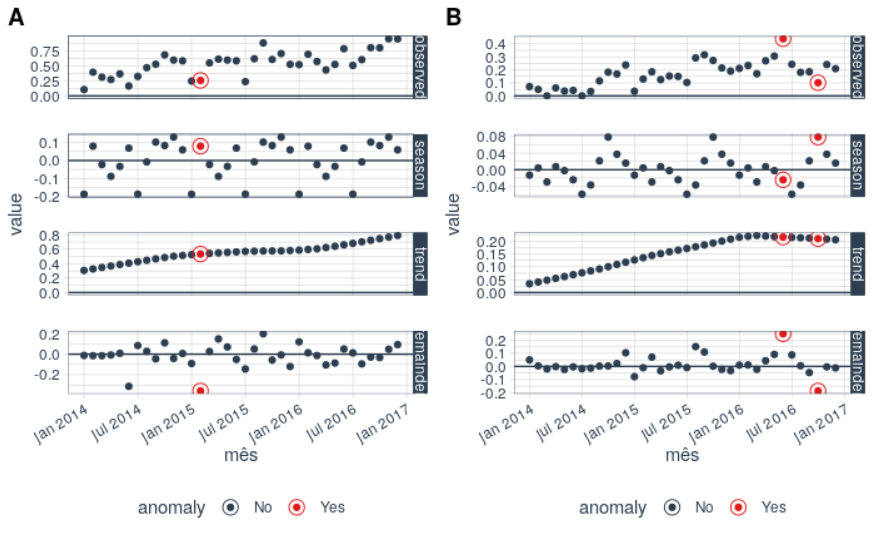


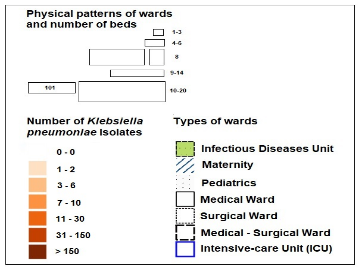

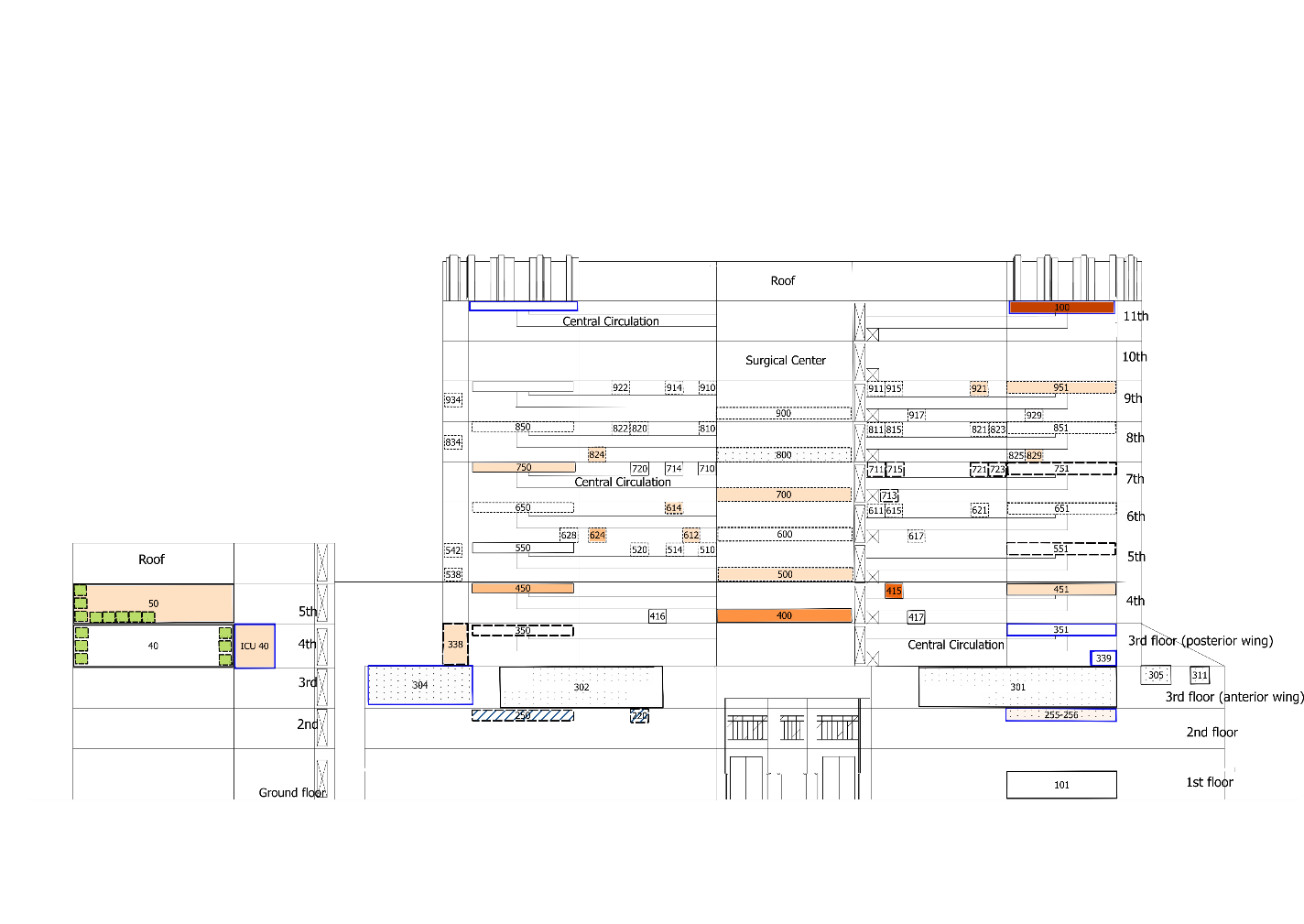


A


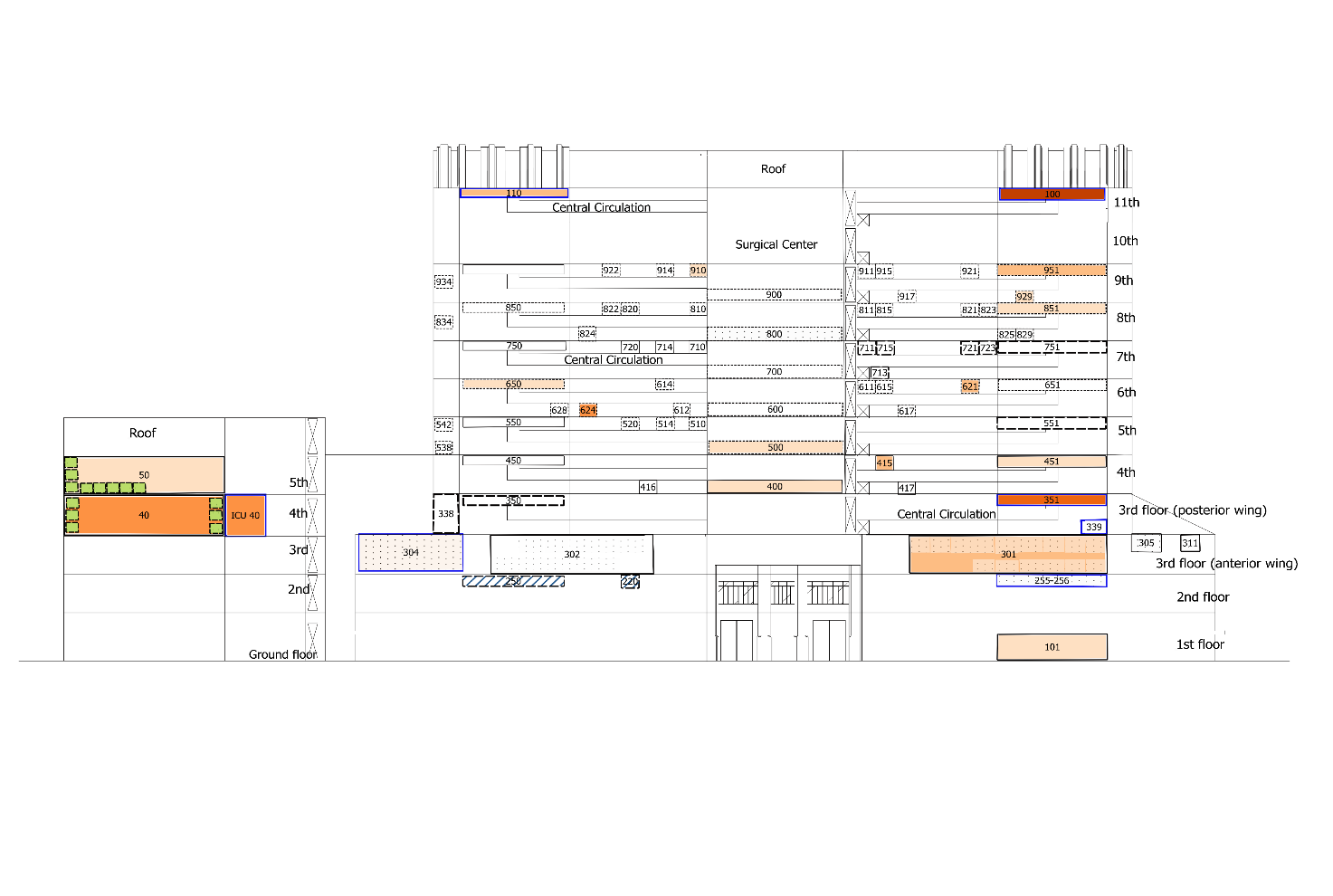


B


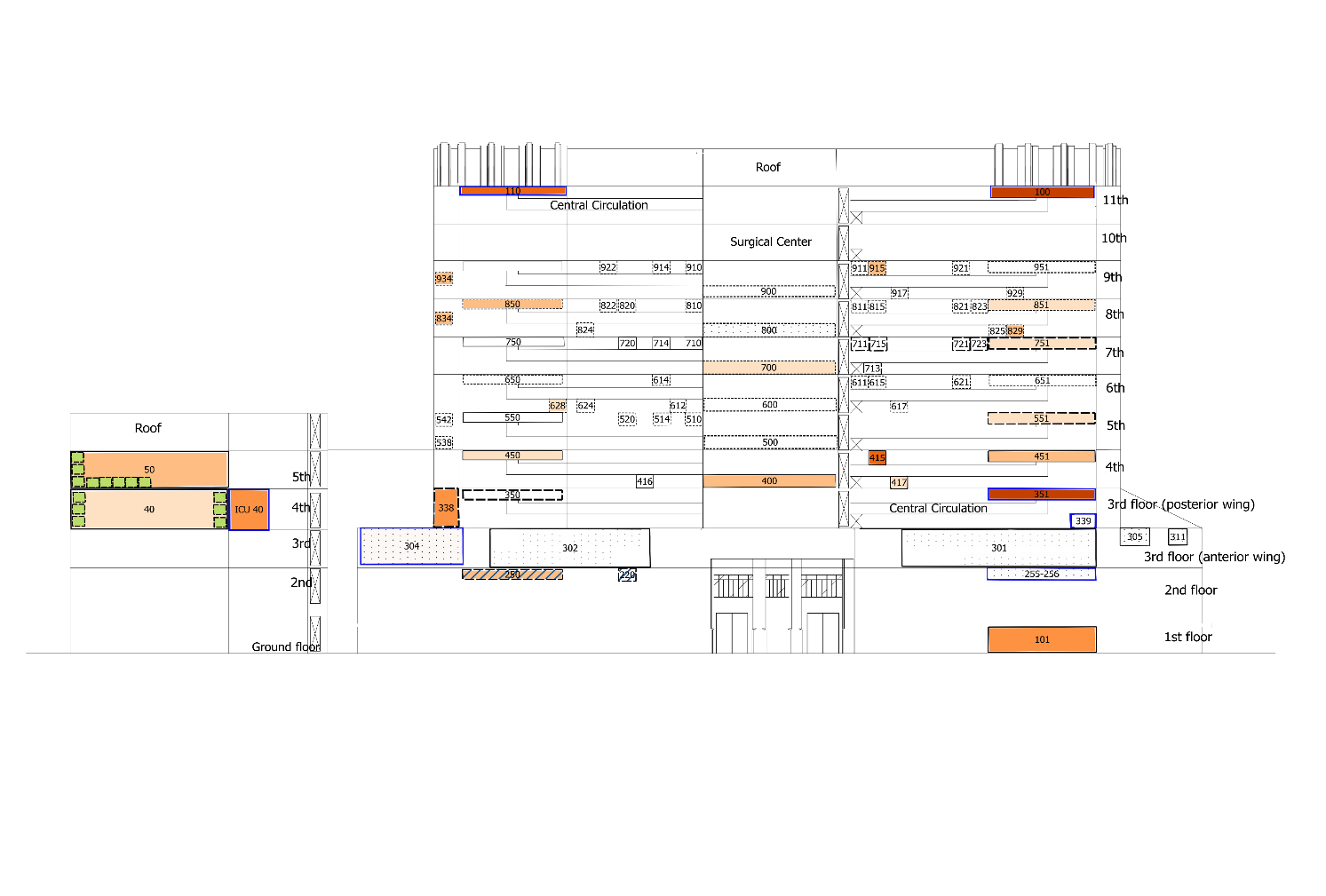


C

**Fig. S9**. Pattern of annual distribution of patients harbouring CRKp complex, regardless of clinical or surveillance material, tertiary federal hospital. **A**: 2014 (n=130 patients); **B**: 2015 (n = 160 patients); C: 2016 (n = 228 patients), p ≤0.001. The darker the orange tone the higher the occurrence

**
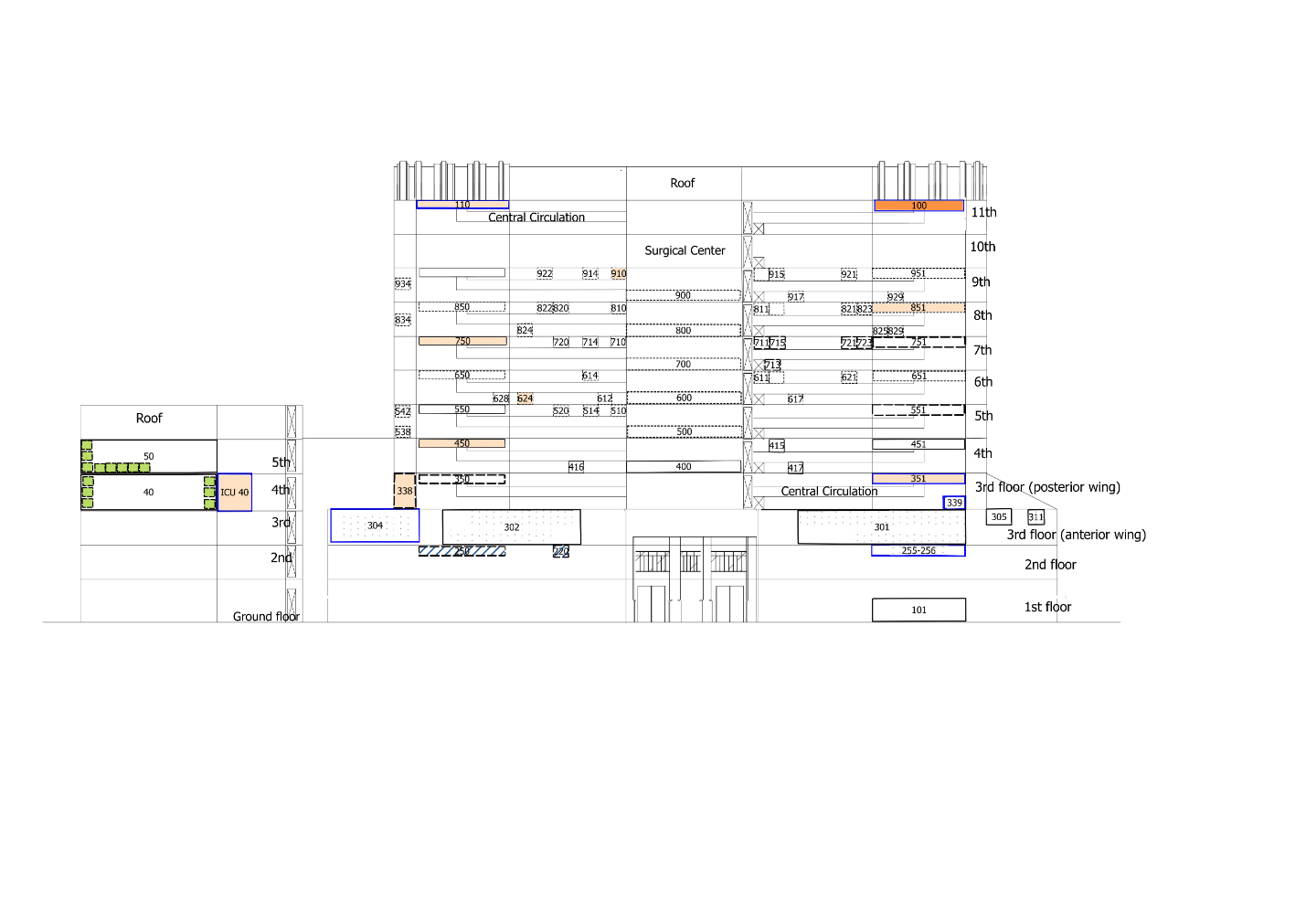

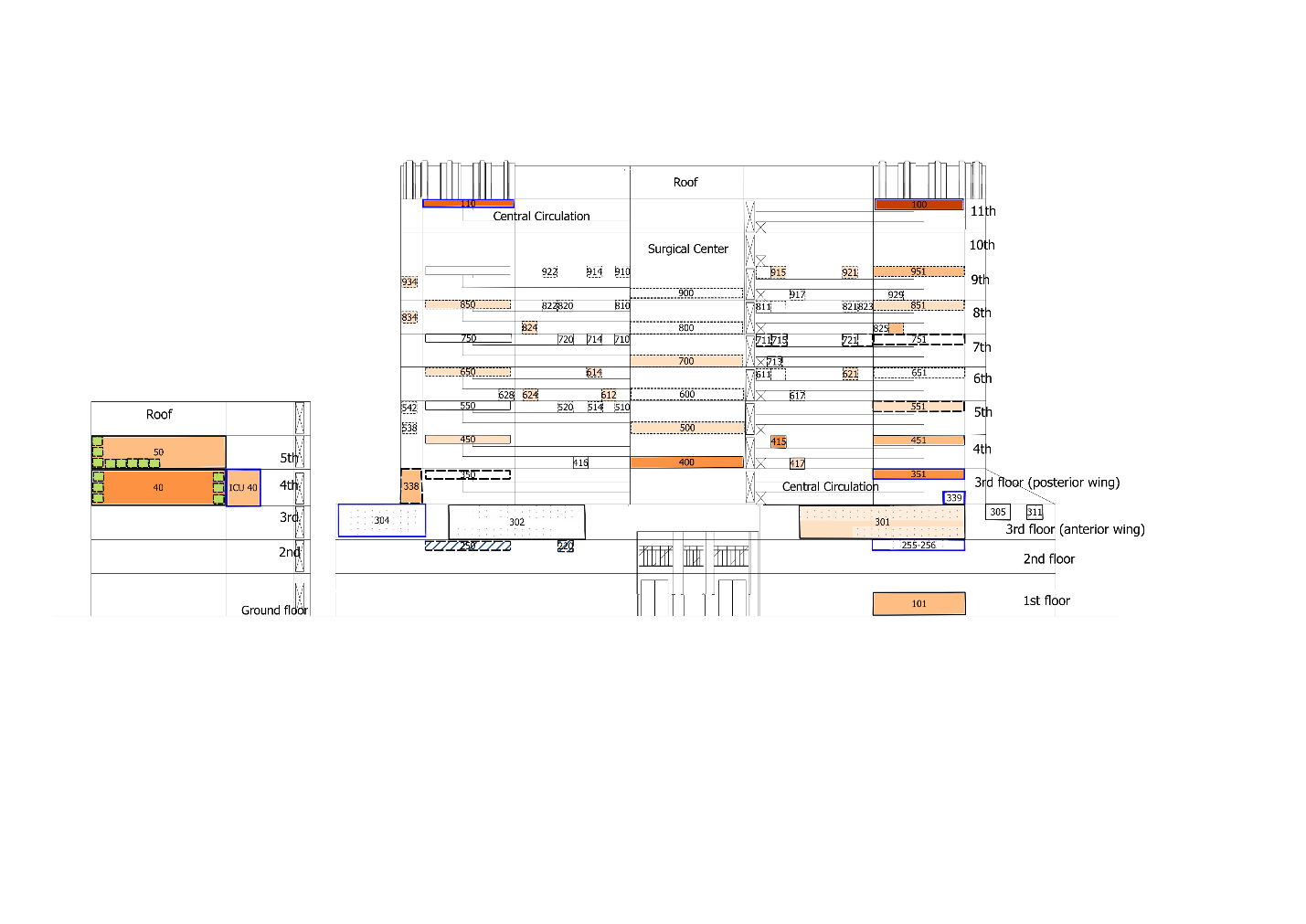

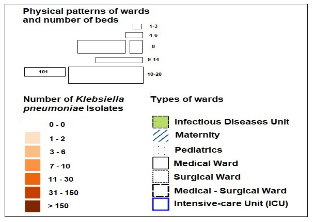

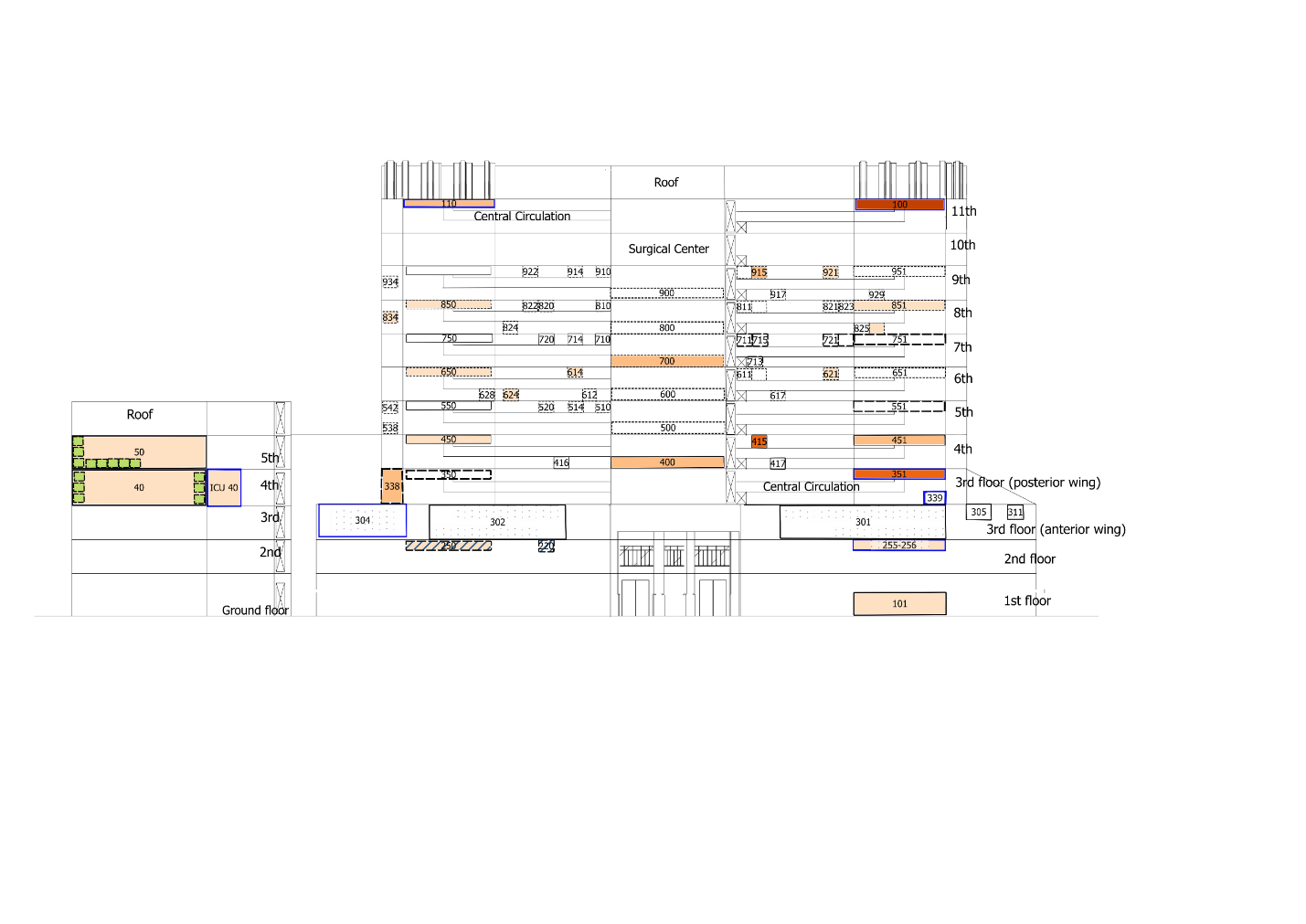
Fig. S10.** Space and time circulation of all patients carrying CRKp complex by ward of admission (n = 202 patient transfers), before (**A**), at the time (**B**) and after(**C**) the detection of CRKp complex colonisation or infection, tertiary federal hospital, 2014-2016. The darker the orange tone, the greater the occurrence

A

B

C

| Ward #:  A 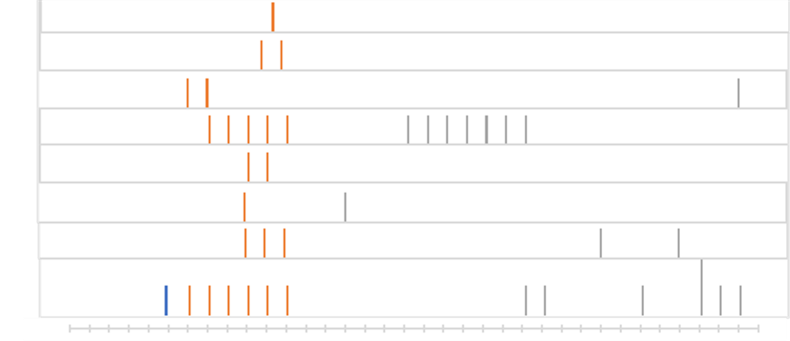 |
| --- |
| 834 |
| 750 |
| 700 |
| 624 |
| 614 |
| 500 |
| 450 |
| 400 |
|  |
|  |
| 551 |
| 451 |
|  |
|  |
|  |
|  |
|  |
| 351 |
| 301 |
|  |
| 101 |

B

5

3 patients

4

2

1

2

Pre cluster Cluster Post Cluster

**Fig. S11**. Number of patients infected or colonised by CRKp complex by clustered wards and month of hospitalization, during the first (**A**) and the second (**B**) pre-cluster, cluster and post-cluster period

**Table S4.** Epidemiological and microbiological characteristics and outcome of patients involved in the first and second cluster

|  | **First Cluster**  (n = 9 patients)^a^ | **Second Cluster**  (n = 15 patients) |
| --- | --- | --- |
| **Age, median (range)** | 66 (33-87) | 71 (0-87) |
| **Male, n (%)** | 5 (56%) | 8 (53%) |
| **Admission Ward of the First Detection** |  |  |
| **An adult ICU, n** | 5 | 8 |
| **Adult medical ward, n** | 3 | 6 |
| **Adult surgical ward, n** | 1 | - |
| **Pediatric ward, n** | - | 1 |
| **Transference between wards, n** | 7 | 13 |
| **First detected in clinical culture, n^b^** | 4 | 9^c^ |
| **First detected in surveillance culture, n** | 5 | 7^c^ |
| **Bloodstream infection, n** | - | 3 |
| **Death from any cause during the hospitalisation, n** | 2 | 6 |

^a^Including the first detected case in pre-cluster period; ^b^First cluster: urine (n=3), secretion (n=1); Second cluster: blood (n=3), urine (n=5), biopsy (n=1); ^c^In one patient the agent was recovered from blood and rectal swabs collected on the same day

9

# 824

# 500

# 614

7

1

1

6

1

1

1

1

2

5

# 450

1

8

1

# 100

# 400

# 700

1

# 750

3

1

4

# 624

6

7

9

10

1

8

3

11

14

1

1

# 951

2

# 301

# 100

1

# 351

# 101

# 451

# 551

# 338

13

# 110

# 550

B

A

1

4

15

12

1

5

1

1

1

1

1

1

1

1

# 350

1

1

# 400

**Appendix Fig.** **12**. Epidemiological link between patients colonised or infected by CRKp complex during the first cluster (**A**), including the index case detected in pre-cluster period (patient #1), and the second cluster (**B**). Each red circle represents the wards involved in the cluster, with the ward number inside. The ward numbers in red are those in the cluster area, while wards with black numbers are those involved by transfer of patients during the cluster periods. Patient number is in blue circle ordered by the date of the first detection, including the first detected case in pre-cluster period of the first cluster (patient #1). The blue arrow represents the opportunity for bacteria transmission between patients, considering the admission ward or unit and the hospitalisation period after the first detection. The dashed blue arrow has the meaning to demonstrate that these units work together as the same ICU. Patients #1 and #8 in the first cluster and patients #2, #14 and #15 in the second group never moved to another ward and, among them, only patient #8 had the direct opportunity to acquire the bacteria from another clustered patient (patient #2) hospitalised in the same period at the same ward (#400).

**Table S5.** Components of AMR surveillance and control program performed for inpatients in the studied hospital, 2014 to 2016.

| 1. Rectal swabs surveillance protocol for target MDR microorganisms, as described in the Appendix Table 2; | |
| --- | --- |
| 1. Daily classification of antimicrobial susceptibility profile of isolates recovered from clinical and surveillance cultures in non-MDR, MDR, possible XDR or PDR (2); | |
| 1. Monitoring of target susceptibility profiles or specific bacterial resistance, such as MRSA, VRE, carbapenem-resistant and polymyxin-resistant Gram-negative bacteria; | |
| 1. Daily notification of target MDR microorganism in all hospital sectors where the patient is allocated, through a standardized form, including the recommendations of contact precautions; | |
| 1. Maintenance of Excel spreadsheets daily, with the names of all patients colonized and/or infected and other information pertinent to AMR, which can be viewed online by health professionals from all sectors of the hospital, including during readmissions; | |
| 1. Daily active surveillance for hospital-acquired infection (VAP, CRBSI, UTI) and of AMR in all ICUs, according to the National Patient Safety Program of ANVISA (4), including incidence density of infections and the use of invasive devices. | |
| 1. Monthly notification of target MDR microorganism and hospital-acquired infection to ANVISA. | |
| 1. Daily analysis and consultation of antimicrobial treatment of patients with positive blood culture results; | |
| 1. Consultation of patients with hospital-acquired infection to guide antimicrobial therapy in all hospital sectors, according to daily demand (request for consultation). | |
| 1. Participation of clinical rounds for optimizing antimicrobial treatments in adult and pediatric ICUs weekly; |  |
| 1. Regular descriptive report (Excel charts, Figures, Tables) of the results of active and passive surveillance of AMR and hospital-acquired infection to all ICUs, including Power-point presentations to the respective ICU healthcare workers and to HICC members; |  |
| 1. Strengthening hand hygiene, environmental cleanliness, contact precaution, cohort of colonised or infected patients, cohort of staff, reassessment of work processes in the affected unit, rectal surveillance, training for the multi-disciplinary team and report of results of the surveillance and infection control program, whenever an outbreak is suspected or confirmed; |  |
| 1. Annual campaign and systematic signaling on hand washing, and measures for the prevention and control of hospital-acquired infection and AMR. |  |

AMR, antimicrobial resistance; ANVISA, National Health Surveillance Agency; CRBSI, catheter-related bloodstream infection; HICC: Hospital Infection Control Committee; ICU: intensive care units; MDR: multidrug resistant; MRSA, methicillin-resistant Staphylococcus aureus; PDR: pandrug resistant; UTI, urinary tract infection; VAP, ventilator-associated pneumonia; VRE, vancomycin-resistant enterococci; XDR: extensively drug resistant.

References

**1.** CLSI. Clinical laboratory Standards Institute. Twentieteh fiftth informational supplement. CLSI document M100-S25. http://www.facm.ucl.ac.be/intranet/CLSI/CLSI-2015-M100-S25-original.pdf. Published 2018. Accessed 16 June 2018.

**2.** Magiorakos AP, Srinivasan A, Carey RB, et al. Multidrug-resistant, extensively drug-resistant and pandrug-resistant bacteria: an international expert proposal for interim standard definitions for acquired resistance. *Clin Microbiol Infect*. 2012;18:268-281. https://doi.org/10.1111/j.1469-0691.2011.03570.x

**3.** P.J. Brockwell RAD. *Time Series: Theory and Methods.* Second ed. New York: Springer; 1991.

**4.** ANVISA. Agencia Nacional de Vigilância Sanitária (Nota técnica Nº 01/2013). Medidas de prevenção e controle de infecções por enterobactérias multirresistentes. <http://portal.anvisa.gov.br/wps/wcm/connect/ea4d4c004f4ec3b98925d9d785749fbd/Microsoft+Word++NOTA+T%C3%89CNICA+ENTEROBACTERIAS+17+04+2013%281%29.pdf?MOD=AJPERES>. Published 2013. Accessed 16 June 2018. <http://portal.anvisa.gov.br/documents/33852/3074175/PNPCIRAS+2016-2020/f3eb5d51-616c-49fa-8003-0dcb8604e7d9>.
