# Supplementary material for: Geographical information system and spatial–temporal statistics for monitoring infectious agents in hospital: a model using Klebsiella pneumoniae complex
Source: Antimicrob Resist Infect Control. 2021 Jun 16;10:92. doi: 10.1186/s13756-021-00944-5 (PMC8207788; doi:10.1186/s13756-021-00944-5)
Supplement: Supplementary file 2 — Additional file 2: Fig S3. Thematic hospital maps of annual detection of K. pneumoniae complex according to the antimicrobial susceptibility profiles and regardless of clinical or surveillance samples. [file 13756_2021_944_MOESM2_ESM.rtf]

Fig. S3. Thematic hospital maps of annual detection of K. pneumoniae complex according to the antimicrobial susceptibility profiles and regardless of clinical or surveillance samples. (A) Non-MDR profile (n = 287 isolates), p <0.008; (B) MDR profile (n = 472 isolates), p <0.008; (C) Possible XDR/PDR profiles (n = 166 isolates), p <0.001; tertiary federal hospital. The darker the orange tone, the higher the occurrence. MDR, Multidrug-resistant; XDR, Extensively-drug resistant; PDR, Pandrug resistant
